# suPAR and Team Nephrology

**DOI:** 10.1186/1741-7015-12-82

**Published:** 2014-05-20

**Authors:** Howard Trachtman

**Affiliations:** 1 NYU Langone Medical Center, Department of Pediatrics, Division of Nephrology, CTSI, 227 E 30th Street, Room #110, New York, NY, USA

**Keywords:** Focal segmental glomerulosclerosis (FSGS), Soluble urokinase receptor (suPAR), Podocyte, B7.1

## Abstract

Primary focal segmental glomerulosclerosis (FSGS) accounts for nearly 10 % of patients who require renal replacement therapy. Elevated circulating levels of soluble urokinase receptor (suPAR) have been identified as a biomarker to discriminate primary FSGS from other glomerulopathies. Subsequent reports have questioned the diagnostic utility of this test. In a study in *BMC Medicine*, Huang *et al*. demonstrate that urinary soluble urokinase receptor (suPAR) excretion assists in distinguishing primary FSGS from other glomerular diseases, and that high plasma suPAR concentrations are not directly linked to a decline in glomerular filtration rate (GFR). This observation suggests that further investigation of suPAR is warranted in patients with FSGS. It should be interpreted in light of a recent report that B7-1 is expressed in the podocytes of a subset of patients with FSGS, and that blocking this molecule may represent the first successful targeted intervention for this disease. These advances highlight the rapid pace of scientific progress in the field of nephrology. Nephrologists should work together, share resources, and expedite the design of protocols to evaluate these novel biomarkers in a comprehensive and scientifically valid manner.

Please see related article http://www.biomedcentral.com/1741-7015/12/81.

## Introduction

There appears to be a basic drive in human beings to try to become members of teams. It is part of our nature as social animals to define ourselves based on our self-perception of who we are and how we differ from those outside our group [1]. Doctors do not change their stripes just because two letters are added to their name after 4 years of medical training, and they are hard-wired, like all people, to join groups. There are large teams such as surgeons, internists, and psychiatrists, and smaller teams such as nephrologists. Within nephrology, there are other sub-identities from which people can choose, such as transplant nephrologist, hypertension specialist, or geriatric nephrologist.

In the past, members of the glomerular team constituted a small cohesive group of specialists who labored hard in the care of patients with glomerular disease. They were fairly unified because there was not too much to splinter the group. The pathophysiology of glomerular disease remained a black box, and therapeutic options were limited. Perceived differences were not sufficient to start a new team.

However, if you have an interest in glomerular disease, then these are very exciting times. Whole exome sequencing, enhanced interaction with other disciplines, improvements in experimental techniques, expanded bioinformatics capacity, and development of systems biology approaches have resulted in dramatic advances in our understanding of how the glomerulus functions in health and disease [2]. A predictable consequence of these striking discoveries is the emergence of teams of devotees who promote the importance of particular mediators of disease or potential therapeutic interventions, to the exclusion of other approaches. That is what being a member of the team means.

The story of the association between elevated circulating levels of soluble urokinase receptor (suPAR) as a potential marker of primary focal segmental glomerulosclerosis (FSGS) is a vivid illustration of this social phenomenon. FSGS is an important cause of glomerular disease in children and adults, and accounts for nearly 10% of patients who develop end stage kidney disease. Moreover, the disease can recur in nearly 25% of patients who undergo kidney transplantation [3]. These clinical features emphasize the compelling need to distinguish FSGS from other forms of glomerular disease. Although suPAR has been recognized for many years as a marker of inflammation and specific infections [4-7], it burst onto the nephrology scene in 2011, when Jochen Reiser’s group reported that a high plasma concentration of this biomarker indicated the presence of primary FSGS versus other forms of primary glomerular disease [8]. suPAR was associated with recurrent glomerular disease after kidney transplantation. Moreover, the molecule appeared to be more than simply an epiphenomenon in FSGS, because *in vitro* studies indicated that suPAR binds to β3 integrin in the podocyte membrane, activates intracellular signaling, promotes foot process effacement, and disrupts glomerular barrier function, leading to proteinuria [7]. This exciting observation was followed by several supportive reports [9-11]. However, over the past year there have been six reports from various cohorts of patients with FSGS and other glomerular diseases indicating that plasma suPAR concentration does not discriminate FSGS from other glomerular diseases, and that the primary determinant of the suPAR level may be the glomerular filtration rate (GFR) [12-17].

### Can suPAR distinguish FSGS from other glomerular diseases?

It is in this context that the report of Huang *et al*. [18] in *BMC Medicine* should be read. In their series of patients with primary glomerular disease, urinary suPAR excretion was significantly higher in patients with primary FSGS (n = 62) compared with all other varieties of glomerular disorders. The cellular variant of FSGS was associated with higher urinary levels than other subtypes, and the excretion correlated with proteinuria in all forms. Urinary suPAR excretion fell in response to effective therapy. The urinary suPAR excretion was biologically active because it induced AP5 expression in cultured human podocytes. The robust findings in this article support the relevance of suPAR as a contributing factor in the pathogenesis of primary FSGS in patients with a preserved GFR. It is consistent with a report that higher urinary suPAR excretion may identify patients with recurrent FSGS in their transplanted kidney [19]. The strengths of this study are the large patient sample, a meaningful approach to distinguishing primary from secondary FSGS based on the extent of epithelial cell foot process effacement, and the incorporation of studies to demonstrate the *in vitro* activity of urinary suPAR. The limitations should be acknowledged, and include a lack of studies to assess stability of suPAR in the urine, an inability to distinguish between various subtypes of suPAR based on processing of the molecule, and lack of long-term follow-up [20]. Nonetheless, the study allows assessment of suPAR to be performed routinely in a readily accessible body fluid that may reflect intra-renal effects more closely than changes in the plasma concentration. It is likely that the members of the suPAR team will be heartened by this report, while the skeptics will express concern about the adequacy of methods used to measure suPAR in the urine and the criteria that were applied to categorize the patients.

### Are other factors causal in FSGS?

There is another exciting recent discovery that is also driving those in the nephrology field to join competing teams. It represents the identification of another factor that might be causal in FSGS, and which could be used both for diagnosis and targets for therapy. Yu *et al*. [21] recently described five patients (four with recurrent FSGS after kidney transplantation and one with FSGS in native kidneys) who were treated with abatacept (cytotoxic T-lymphocyte–associated antigen 4–immunoglobulin fusion protein [CTLA-4–Ig]) based on positive staining for B7-1 in glomerular podocytes. All five patients experienced a clinically significant reduction in proteinuria. This has prompted a surge in the abatacept club membership. However, within 4 months, two brief reports have appeared questioning the validity of these findings, and suggesting that the B7-1 pathway is not active in glomerular disease and is not a therapeutic target [22,23]. The community is splitting up into teams: those who consider suPAR, B7-1, or other favorite candidates to be the main causal factor for FSGS.

## Conclusions

The current position of suPAR in the setting of glomerular disease has been succinctly summarized in a recent, balanced editorial commentary [24]. Editorials on B7-1 will follow in short order. Rather than simply rehearse arguments that have already been made in the literature, I will address the following open question: will the pressure to join nephrology teams interfere with a rational and comprehensive evaluation of these new pathways in the pathogenesis of FSGS and other glomerular diseases? What is the thoughtful nephrologist to do in this challenging era of enormous growth in the knowledge base for glomerular disease? I suggest we should applaud the innovative efforts of clinical scientists such as Jochen Reiser and Peter Mundel, who are uncovering novel pathways in the development of kidney disease. It would seem prudent to maintain the glomerular disease team as a large inclusive club that welcomes all comers. Membership should be defined by a commitment to rigorously study newly identified biomarkers and validate disease targets in well-designed clinical trials. The efforts of investigators such as Huang should be analyzed carefully by Team Nephrology, and added to the mix as the community works to clarify the value and utility of new discoveries like suPAR and B7-1. Fortunately, the scientific advances are being reinforced by the creation of a stable infrastructure to promote ongoing evaluation of new biomarkers and novel therapies for glomerular disease. The National Institute of Diabetes and Digestive and Kidney Diseases (NIH-NIDDK) has demonstrated its strong commitment to improving the outcomes for patients with primary glomerular disease by investing vital resources in establishing the Nephrotic Syndrome Study Network (NEPTUNE) and Cure Glomerulonephropathy (CureGN) prospective cohort studies [25]. Research in glomerular disease is moving forward on all fronts. For nephrologists, Charles Dickens had it wrong. It is the best of times, it is an age of wisdom, it is a season of light, it is a spring of hope, and we have everything before us.

## Competing interests

No competing interests to report.

## Authors’ information

HT author is a participant in the NEPTUNE and CureGN observational cohort studies, and has been the principal investigator for clinical trials in FSGS.
